# Extracellular Matrix Remodeling of the Umbilical Cord in Pre-eclampsia as a Risk Factor for Fetal Hypertension

**DOI:** 10.1155/2011/542695

**Published:** 2010-12-29

**Authors:** Lech Romanowicz, Zofia Galewska

**Affiliations:** Department of Medical Biochemistry, Medical University of Białystok, ul. Mickiewicza 2, 15-089 Białystok, Poland

## Abstract

The human umbilical cord forms a connection between the placenta and the foetus. It is composed of two arteries and one vein surrounded by Wharton's jelly. Pre-eclampsia is accompanied by extensive remodeling of extracellular matrix of umbilical cord. Matrix metalloproteinases (MMPs) are engaged in degradation of extracellular matrix proteins and activation/inactivation of certain cytokines and enzymes. These enzymes will probably play a central role in the release of matrix-embedded cytokines and growth factors. MMP-2 (gelatinase A) is the main collagenolytic enzyme of both umbilical artery and vein. Other metalloproteinases are present in several times lower amounts. Reduced activity of collagen-degrading enzymes may be a factor, which enhances the accumulation of collagen and some other proteins in the pre-eclamptic umbilical cord tissues. It seems to be possible that similar alterations occur in other fetal blood vessels. It may result in an increase in peripheral resistance as well as an increase in the blood pressure in the fetal vascular system. Some observations suggest that the raised pressure may persist after birth. Pre-eclampsia may be a factor that evokes an initiation of hypertension in utero and its amplification through childhood and adulthood.

## 1. Structure of the Umbilical Cord

The human umbilical cord forms a connection between the placenta and the foetus. It is composed of three blood vessels of different structure and function, one vein, which transports oxygenated and nutrition-rich blood from placenta to foetus, and two arteries, which transport deoxygenated blood and metabolic waste products from foetus to placenta. All these vessels are surrounded by Wharton's jelly [1], which constitutes the major part of human umbilical cord and provides a thick protective mantle around vessels. Wharton's jelly plays also an important role as a storage for some compounds, such as growth factors [2]. 

The extracellular matrix (ECM) in the vascular wall contains many macromolecules (collagen, elastin, proteoglycans, and glycoproteins) necessary for the structural and functional properties of vessel wall [3]. Proteolysis is a major process leading to changes in the ECM [4].

## 2. Remodeling of the Umbilical Cord in Pre-eclampsia

### 2.1. Umbilical Cord Artery

It was found in our studies that pre-eclampsia is accompanied by an extensive remodelling of the ECM of the umbilical cord. The umbilical cord arteries (UCAs) of newborns delivered by mothers with pre-eclampsia contain more than twice the amount of collagen and markedly less elastin in comparison to the corresponding arteries of newborns delivered by healthy mothers [5]. The changes in collagen composition are accompanied by an early reduction of hyaluronic acid and its replacement by sulphated glycosaminoglycans [6]. Pre-eclampsia-associated decrease in hyaluronic acid content is a result of a decreased synthesis of this substance, whereas an increase in sulphated glycosaminoglycans content is rather a result of slower degradation of newly synthesized these compounds [7]. Significant reduction in the activities of neutral endoglycosidases degrading chondroitin 4-sulphate and 6-sulphate, dermatan sulphate and heparin was found. The activities of main exoglycosidases (N-acetylhexosaminidase, *β*-galactosidase, *α*-iduronidase, and *β*-glucuronidase) also decrease [8]. Furthermore, the increase in the ratio of the content of sulphated glycosaminoglycans to the content of the protein core of proteoglycans of newborns delivered by mothers with pre-eclampsia was determined. The expression of decorin core protein was enhanced, whereas biglican expression was lowered. The high-molecular proteoglycans, for example, versican and perlecan obtained for pre-eclamptic UCAs were less prominent than in the case of control material [9]. The pre-eclampsia-associated alterations in proteoglycans of the UCAs seem to participate in the rearrangement of the ECM of this tissue. Along with the pathomorphological and biochemical changes, they may affect the mechanical properties of the UCAs and disturb fetal blood circulation [10].

An increase in tissue collagen content may result from increased synthesis, decreased degradation, or a combination of both phenomena. It was found by metabolic studies (“pulse-chase” experiment) with the use of 14C-proline that tissue slices of the UCAs taken from newborns delivered by mothers with pre-eclampsia incorporate much more of this amino acid into collagenase-sensitive and hydroxyproline-containing protein and demonstrate decreased degradation of newly synthesised collagen in comparison to corresponding slices taken from newborns delivered by healthy mothers [11]. Also pre-eclamptic serum stimulates collagen synthesis by human skin fibroblasts growing in vitro [12]. These observations allow concluding that the pre-eclampsia-associated increase of collagen content in the UCAs results from both enhanced synthesis and decreased degradation of newly synthesised collagen. It is known that the activity of collagenolytic enzymes is a main factor regulating collagen degradation rate in tissues. 

Arterial hypertension seems be a factor which affects collagen metabolism in the UCA wall [13–15]. Such a combination of these two phenomena (induced by hypertension) was described by Bishop et al. [16]. They found that the experimentally induced pressure overload in the pulmonary artery of rabbits (reducing the diameter of this artery by 50%) results in the right ventricular hypertrophy accompanied with a six-fold increase in collagen synthesis and a decrease (by half) in collagen degradation rate in the right heart ventricle. It resulted in more than a two-fold accumulation of collagen in myocardium after 14 days of the experiment. In the same time, no significant change was observed in the left ventricle. It seems possible that pre-eclampsia-associated increase of collagen content in the UCA may be a result of a mechanism similar to that described by Bishop et al. [16]. An increase of blood pressure in the UCA may enhance the biosynthesis of collagen and decrease its degradation. On the other hand, the remodelling of the UCAs may be responsible for the decrease in the fetuses blood flow of women with pre-eclampsia.

Pre-eclampsia-induced accumulation of collagen is accompanied by a distinct reduction in elastin content in the UCA wall [17]. The replacement of elastin by collagen may decrease the compliance of the umbilical cord arteries. The reduction in compliance can increase the peripheral resistance, and, inversely, this increased resistance may modify the umbilical vessels elastin components. It seems to be possible that similar alterations occur in other fetal blood vessels. It may result in an increase of peripheral resistance as well as an increase of the blood pressure in the fetal vascular system. Some observations suggest that the raised pressure may persist after birth [18]. One may expect that the pre-eclampsia associated changes in arterial walls may affect the vascular system in adulthood. Pre-eclampsia may be a factor which evokes an initiation of hypertension in utero and its amplification through childhood and adulthood [19].

These phenomena correspond to an early ageing of these tissues [5, 20]. The accumulation of collagen with simultaneous decrease in elastin content in the umbilical cord arteries may reduce the elasticity of arterial wall and decrease the blood flow in the fetus of woman with pre-eclampsia.

### 2.2. Umbilical Cord Vein

We have also found that pre-eclampsia is accompanied by an extensive remodeling of the extracellular matrix of the umbilical cord vein (UCV) of newborns delivered by mothers with pre-eclampsia. It contains less collagen in comparison to the corresponding veins of newborns delivered by healthy mothers [21]. Furthermore, the change in relationship among collagens of main types was detected. Pre-eclamptic UCVs contain less type I collagen and more type III collagen. That polymeric collagen is less susceptible to the action of depolymerizing agents [22]. Although the elastin content is higher in pre-eclamptic UCV, its polymerization is lower [21]. The amounts of the core proteins of decorin, biglycan, and versican were distinctly higher in pre-eclamptic veins in comparison to control vessels [23]. The content of glycosaminoglycans did not change significantly [24], but specific activities of neutral endoglycosidases (degrading chodroitin 6-sulphate, dermatan sulphate, heparin, heparan, and keratan sulphate) and main exoglycosidases (N-acetylhexosaminidase, *β*-galactosidase, and *β*-glucuronidase) decreased in pre-eclamptic vessel [25].

### 2.3. Wharton's Jelly

The great remodeling of pre-eclamptic Wharton's jelly ECM has been also found. The higher total content of collagen with its solubility decreased has been detected. The lower solubility of collagen in pre-eclamptic tissue may be evoked by a relatively higher proportion in the amount of type III collagen. Pre-eclamptic Wharton's jelly contains higher amounts of glycosaminoglycans in comparison to control tissue. Premature replacement of hyaluronic acid by sulphated glycosaminoglycans has been observed [26]. Pre-eclampsia is associated with a lower level of all proteoglycan core proteins, especially those of higher molecular mass (such as versican) although the same set of core proteins were found in normal and pre-eclamptic Wharton's jelly. The higher ratio of sulphated glycosaminoglycans to proteoglycan core proteins has been detected in this tissue [27]. It can be a result of decrease in their degradation, because of the lower activity of neutral endoglycosidases (degrading chondroitin 4- and 6-sulphate, dermatan sulphate, heparin, and heparan sulphate) and two exoglycosidases of main significance (N-acethylhexosaminidase and *β*-galactosidase) [28].

## 3. Proteolytic Activity of the Umbilical Cord

### 3.1. Umbilical Cord Artery

It seems that the remodelling of the umbilical cord vessels may be responsible for the decrease of blood flow in the foetus of women with pre-eclampsia [23, 29, 30]. On the other hand, a reduction of blood flow in the fetal circulation may be responsible for remodeling of the umbilical cord vessels. Generally, a reduction of maternal blood flow to the uterus rather than fetal blood flow (unless the fetus is small) has been observed in pre-eclampsia and/or intrauterine growth restriction (IUGR) [31, 32]. We evaluated whether or not this phenomenon is associated with alterations in the activities of collagenolytic, gelatinolytic, and nonspecific proteolytic enzymes that may be involved in collagen degradation. The activity of prolidase that provides proline as a substrate for collagen biosynthesis was also investigated.

It is apparent from our studies [13] that the extracts of both control and pre-eclamptic UCAs demonstrate significant proteolytic activities, especially at neutral pH range. Albumin is readily digested by UCA-proteases to low-molecular-mass products. 

Neither the control nor the pre-eclamptic UCAs extracts digested native collagen. Treatment of reconstituted collagen fibres with UCA-extracts did not affect the electrophoretic mobility of collagen subunits. Treatment of UCA-extracts with latent collagenase-activating agents (trypsin, p-chloromercuric benzoate) did not evoke an appearance of collagenolytic activity. Native collagen was not degraded in the experimental conditions [13].

In contrast to that, gelatin (denatured collagen) was digested to low-molecular-mass products by UCA-extracts. The gelatinolytic activity of crude UCA-extracts was very low, but it increased many times under the action of trypsin. It can be concluded that the arterial wall tissue contains a latent form of gelatinase which may be activated in a tissue by other proteolytic enzymes. Pre-eclampsia is associated with a distinct decrease of gelatinolytic activity in UCA-wall [13].

Degradation of collagen may occur both intracellularly (before its secretion) or extracellularly. The “pulse chase” experiments performed by Bienkowski et al. [33] demonstrated that the amount of intracellularly degraded collagen attained a value equal to approximately 20% of the amount synthesised during the labelling period. Similar observations were reported by Andersson and Warburton [34]. Extracellular collagen may be internalized by phagocytosis of collagen fibrils and degraded by some lysosomal proteinases [35].

Interstitial collagens, the most abundant protein of connective tissue, are degraded by specific collagenolytic enzymes. Products of collagenolysis denature at physiological temperature and become susceptible to the action of gelatinases and other nonspecific proteases.

### 3.2. Umbilical Cord Vein

A significant role in collagen degradation in the UCV wall is attributed to gelatinase A (MMP-2) [36, 37]. That enzyme is capable of cleaving soluble, triple helical type I collagen. It is of interest that zymography demonstrates the presence of gelatinolytic activity in control UCV extracts, even those not treated with p-aminophenylmercuric acetate (APMA). The gelatinolytic activity observed on the zymogram may be a result of the activating action of sodium dodecyl sulphate (SDS) on the latent form of gelatinase A [38]. The treatment of progelatinases with APMA causes a conformational alteration that allows a stepwise autolytic cleavage of the propeptide from latent enzymes [38]. Such cleavage results in the activation of progelatinases. The crude extracts of control UCV contain only a trace of actual collagenolytic activity, but its potential activity is distinctly visible. The action of trypsin on control UCV extract resulted in an increase in the degradation of reconstituted collagen fibres at least 7 folds. In contrast to these findings in control material, it was not able to detect either actual or potential collagenolytic activity in the pre-eclamptic vein [29].

The actual gelatinolytic activity of crude UCV extracts was low, but potential activity (induced by trypsin) is distinctly apparent. This allows us to conclude that the venous wall tissue contains a latent form of gelatinase, which may be activated by tissue proteolytic enzyme(s). One maximum of activity observed at pH 7.5 seems to correspond to gelatinase A, which was detected by zymography. Its activity was lower in the pre-eclamptic vein. The other maximum of activity was detected in the slightly acidic pH range (pH 6.0–6.5). This must be due to a different enzyme or mixture of enzymes. Pre-eclampsia is associated with a distinct decrease in the activity of this/these enzyme/s. Albumin is readily digested by UCV-proteases to low-molecular-mass products. The extracts of control UCVs demonstrate significant actual and total proteolytic activities, especially within a neutral pH range. It is of interest that both the actual and total proteolytic activities of pre-eclamptic UCV extract, at a neutral pH range, were distinctly lower in comparison to the control [29]. These nonspecific proteases may contribute to the degradation of collagenolysis products to low molecular peptides.

It is not possible to say whether the lower potential activities of collagenolytic and gelatinolytic enzymes, as well as those of nonspecific proteases, may promote an accumulation of collagen in the pre-eclamptic UCV wall. In our earlier studies, we observed a reduction of collagen content in pre-eclamptic umbilical cord veins [21].

The latent forms of proteolytic enzymes do not probably contribute to collagen degradation in the investigated veins. Two phenomena of the mechanism of reduction in the potential activities of the investigated enzymes should be taken into account. One of them may be a toxic effect of pre-eclampsia on fibroblasts or other cells [39]. The other may be an increase in Tissue Inhibitor of Metalloproteinases (TIMPs) that reduce the collagenase, gelatinase, and prolidase as well as nonspecific proteinase activities [40–42].

It seems that pre-eclampsia-associated decrease of collagen content in the pre-eclamptic vein may be evoked by a reduction in collagen biosynthesis. This reduction may be connected with low prolidase activity in the pre-eclamptic veins [29]. Prolidase [E.C.3.4.13.9] is a cytosolic exopeptidase, cleaving imidodipeptides with C-terminal proline [42, 43]. The biological function of this enzyme involves the degradation of collagen peptides and the recycling of proline from imidodipeptides for collagen resynthesis. According to Jackson et al. [44], the efficiency of proline recycling from imidodipeptides is about 90%. It is possible that an absence of prolidase severely impedes the efficient recycling of collagen proline. Prolidase may regulate the turnover of collagen and may be a rate-limiting factor in the regulation of collagen production. The low activity of prolidase in the pre-eclamptic vein may be a factor which reduces collagen biosynthesis by fibroblasts contained in this vein.

### 3.3. Wharton's Jelly

It can be seen from the our results that the collagen content is higher in pre-eclamptic Wharton's jelly than in control tissue when calculated per gram of fresh, wet tissue [45]. Such a phenomenon disappears after dehydration of Wharton's jelly of both origins [6, 46–48]. The differences are probably associated with various hydration degrees as a consequence of different amounts of hyaluronic acid that is a strongly hydrophilic substance. Wharton's jelly of newborns delivered by mothers with pre-eclampsia contains less hyaluronic acid therefore; it binds less water than the control tissue [46–48]. For this reason, the proportional amount of collagen in pre-eclamptic Wharton's jelly is significantly higher.

The gelatinolytic activity in control and pre-eclamptic samples of Wharton's jelly, measured by the assay of hydroxyproline in gelatine degradation products, is very low. The action of trypsin results in a significant increase in this activity. The effect of trypsin was similar in both tissues. The control extracts demonstrate two peaks of gelatinolytic activity in a wide pH range, whereas the pre-eclamptic tissue demonstrates one peak only at pH 7.0. Zymographic analysis of control and pre-eclamptic Wharton's jelly demonstrates different electrophoretic patterns of gelatinolytic enzymes. The zymography allowed to evaluate both the active and latent forms of gelatinases, which differ in molecular weights (about 10 kD). Control Wharton's jelly contains two latent forms of gelatinolytic enzymes: gelatinase A (MMP-2, 72 kD) and gelatinase B (MMP-9, 92 kD). The trace 62-kD band is associated with an active form of gelatinase A. In contrast to control tissue, the main gelatinolytic enzyme of pre-eclamptic Wharton's jelly is gelatinase A (MMP-2) [45] of the molecular weight about 72 kD [38, 49, 50]. Prolidase from control Wharton's jelly extracts releases about 40 nmol of proline per minute per milligram of protein. Pre-eclampsia does not affect prolidase activity in Wharton's jelly [45].

The weak gelatinolytic activity measured by the assay of hydroxyproline released from gelatine allows conclusion that Wharton's jelly contains mainly latent forms of gelatinase. They may be activated in this tissue by other proteolytic enzymes. The action of trypsin resulted in a significant increase in gelatinolytic activity, which seems to result from conversion of latent gelatinases into an active enzyme [38, 51].

It is apparent from our studies [45] that the extracts of both control and pre-eclamptic samples of Wharton's jelly demonstrate significant proteolytic activities, especially at neutral pH. This activity distinctly increases after pretreatment with trypsin. Proteolytic enzymes are synthesized in proenzyme forms. Their conversion into active enzymes requires a limited proteolysis of inactive precursors. Such an effect may be achieved in vitro by the action of trypsin [13, 51].

The distinct reduction in gelatinase activity in pre-eclamptic Wharton's jelly can be elucidated that gelatinase forms an inactive complex with one of TIMPs. Such a complex dissociates under the action of trypsin or sodium dodecyl sulphate. The decrease in gelatinolytic activity of Wharton's jelly may be a factor that reduces the breakdown of collagen and promotes an accumulation of this protein [13, 47, 52].

### 3.4. Matrix Metalloproteinase-1

The UCA wall contained MMP-1 (collagenase 1). Control UCA extracts demonstrated low amount of that enzyme, only about 6 mg per kg of protein. Pre-eclampsia was associated with almost two-fold decrease in MMP-1 (collagenase 1) content in comparison to control UCA. The actual activity of MMP-1 (collagenase 1) in control UCA extracts was low, and in pre-eclamptic UCAs it was twice lower. Activation with trypsin allowed us to determine total MMP-1 activity. The UCA extracts (control and pre-eclamptic) treated with trypsin demonstrated an increase in MMP-1 activity. Total MMP-1 activity in pre-eclamptic UCAs extracts was about 30% lower than in control artery extracts. The effect of trypsin on the pre-eclamptic extract resulted in two-fold increase in MMP-1 total activity in comparison to its actual activity. But even such an increase in collagenase 1 activity in pre-eclamptic UCAs did not permit to reach the activity observed in control UCAs [53].

The comparison of content, actual (nonactivated) and total (activated with trypsin) specific activities of MMP-1 in UCA wall slices, incubated in the medium with control UC serum or pre-eclamptic UC serum, indicated that pre-eclamptic serum exerted an inhibitory effect on content and activity of collagenase-1 in that tissue. High concentration of TIMP-1 in pre-eclamptic-serum-treated tissue (and in lesser degree TIMP-2) might be one (probably the main) factor, which inhibited MMP-1 activity [54].

MMP-1 was presented also in the UCV wall at low amount. Control material contained about 4 mg of collagenase 1 per kg of protein. Pre-eclampsia was accompanied with 30% increase in its content. The actual activity of MMP-1 in both control and pre-eclamptic UC vein extracts was similar to that of UCAs. Action with trypsin did not change the activity of collagenase 1. It suggested that the whole enzyme was in active form in control UCV. Pre-eclamptic material demonstrated 20% higher total activity of MMP-1 in comparison to control (data not published).

### 3.5. Gelatinases (MMP-2 and MMP-9)

MMP-2 (gelatinase A) and MMP-9 (gelatinase B) are metalloproteinases that digest denatured collagen to low molecular products [55]. Both collagenases and gelatinases may be inhibited by the action of TIMPs. Aimes and Quigley [56] found that gelatinase A, free of TIMPs is capable of cleaving soluble, triple helical type I collagen generating the 3/4 and 1/4 length collagen fragments characteristic of vertebrate interstitial collagenases. Both gelatinases participate in degradation of other ECM components like proteoglycans, elastin, and structural glycoproteins. Furthermore, MMP-2 can convert pro-MMP-9 into active enzyme [57]. A significant role in collagen degradation is attributed to MMP-2 (gelatinase A) [37, 56].

#### 3.5.1. MMP-2

The content of MMP-2 in UCA extracts were the highest in comparison to other metalloproteinases. Control material contained 75 mg MMP-2 per kg of protein. Pre-eclampsia was associated with a distinct decrease in the amount of this enzyme. It was 48 mg of total MMP-2. Determination of the active form of gelatinase A indicated that pre-eclampsia induces a decrease in the amount of active form of that enzyme from 11 mg/kg protein in control UCA extract to about 8 mg/kg protein. However, there was no difference in percentage of active form in relation to total MMP-2 content (15% in control and 16% in pre-eclamptic UCA extract) [53]. 

The high amount of total gelatinase A was detected—about 55 mg/kg of protein in the extracts from the UCV wall of newborns delivered by healthy mothers. Pre-eclampsia was associated with a decrease in MMP-2 total content—to about 38 mg/kg of protein. Active form of that enzyme was presented in less than 10% of total amount. UCV wall of control newborns contained about 4 mg of active MMP-2 and pre-eclamptic venous wall contained about 2 mg per kg of protein. The percentage of active form in relation to total MMP-2 content was twice lower in UCV in comparison to UCA. It was 8% in control material and 6% in pre-eclamptic tissue. MMP-2 is the main collagenolytic enzyme in UCVs wall [58].

Gelatin (denatured collagen) was evidently degraded by UCA extracts at a wide pH range. The zymogram demonstrated the presence of gelatinolytic enzyme(s) in the extracts of both control and pre-eclamptic UCAs. It was of interest that such an activity was clearly apparent, even in a crude UCA extract, nontreated with any activating agents. The control UCA extract demonstrated two lysis bands. The upper band (latent form of MMP-2) was distinctly visible. The lower one (active form of MMP-2) was faint. The activation of control UCA extract with APMA did not change the electrophoretic mobility of gelatinolytic enzyme(s), but it was observed a partial loss of upper band and increase in intensity of lower band. Such a change indicated partial degradation of latent form of the enzyme to the active form with lower molecular mass. Pre-eclamptic nonactivated extract and extract activated with APMA showed a similar pattern as the control [53].

Similar results were observed for control and pre-eclamptic UCV extracts nonactivated and activated with APMA. However, the activating effect of APMA was distinctly lower for pre-eclamptic venous extract in comparison to control [29].

### 3.6. Matrilysins (MMP-7 and MMP-26)

MMP-7 can degrade insoluble elastin and other vascular basement membrane components. This enzyme participates in activation of different MMPs [59]. Human pro-MMP-1 and -2 and can be activated by MMP-7.

MMP-26 is an enzyme with higher specificity than MMP-7. MMP-26 selectively cleaved type I gelatine and *α*1-proteinase inhibitor [60]; however, it did not digest collagens, laminin, elastin, *β*-casein, plasminogen, or soybean trypsin inhibitor [61]. The release of MMPs from endothelial cells may be regulated by variety of factors, for example, fibronectin, vascular endothelial growth factor (VEGF), and other cytokines [60]. The proteolytic activity of MMPs is precisely regulated by the action of zymogen—activating enzymes and antiproteases. Both MMP-26 and MMP-7 can activate MMP-9 but their cleavage sites in pro-MMP-9 are different. MMP-26 do not cleave pro-MMP-2; another gelatinase, indicating that pro-MMP-9 activation by MMP-26, is highly selective [62]. These two MMPs show an increased expression in a variety of tumours and other pathologically altered tissues [59, 60].

#### 3.6.1. Matrilysin-1 (MMP-7)

Human umbilical cord tissues contained MMP-7. Free form of that enzyme was detected in UCA and UCV walls extracts. Indicated molecular weight of (28 kDa) corresponds to free precursor form of this enzyme. Furthermore, high molecular weight complexes containing that metalloproteinase were detected in all UC tissues. The action of dithiothreitol resulted in a release of several compounds reacting with anti-MMP-7 antibody with lower molecular weights, but no free forms of MMP-7 were detected in control and pre-eclamptic Wharton's jelly [63]. Those compounds may be complexes of this enzyme with other ECM components.

Immunoenzymatic assay indicated that pre-eclampsia was accompanied by a significant increase of MMP-7 content in UCA and UCV walls. There is not change of MMP-7 content in pre-eclamptic Wharton's jelly in comparison to control subjects. Zymographic analysis demonstrates a detectable activity of MMP-7 in all control and pre-eclamptic UC tissues at positions corresponding to complex form of this enzyme with other components. It was not found any casein degradation at position for free active or latent form of described matrilysin. Clear band at position of 54.3 kDa corresponds to complex of MMP-7 with probably TIMP-1 or TIMP-2. The band with higher molecular mass (about 94.9 kDa) shows proteolytic activity of MMP-7 complex with other ECM components [63].

### 3.7. Membrane-Type Matrix Metalloproteinases (MT-MMPs)

It was found that UC vessels and Wharton's jelly of control and pre-eclamptic newborns contained MT-MMPs. Quantitative assays of MT-MMPs showed significant differences of their amount in pre-eclamptic UC components. The content of respective MT-MMP in control tissue was equal to 100%. The amount of MT-1 and MT-2 in pre-eclamptic UCA increased to 174% and 309% of control tissue, respectively. There was not change of MT-5 content in that tissue (107% of control). Pre-eclamptic UCAs contained less amount of MT-3, only 52% of control. Pre-eclampsia was associated with an increase in MT-1, MT-2, and MT-5 content in UCV (157%, 217%, and 240% of control, resp.). Only amount of MT-3 was reduced to 60% in pre-eclamptic vein. Significant increase in MT-2 and MT-3 content of pre-eclamptic Wharton's jelly was found (183% and 299% of control, resp.). The amount of MT-5 was almost the same, 94% of control. MT-1 content was decreased in pre-eclamptic Wharton's jelly to 53% of control (data not published).

Western immunoblot experiments showed that human UC tissue extracts reacted with anti-MMP-14 and anti-MMP-15 antibody. Free form of both investigated MMPs was detected in all investigated tissue extracts. Indicated molecular weight of them (about 54 kDa) corresponds to free active form of respective MMP [57]. Furthermore, high molecular weight complexes containing these metalloproteinases were detected in all UC tissues, with molecular weight about 200 kDa, 115 kDa, and 95 kDa. They could be complexes of respective MT-MMP with TIMPs and some ECM components. It is of interest that the action of reducing agent on these complexes resulted in a decrease in their molecular weights. The bands with high molecular mass (200 kDa and 115 kDa) disappeared. The band of the molecular weight 56 kDa reacting with anti-MMP-14 antibody was more intensive. A band corresponding to free MMP-15 disappeared. The action of dithiothreitol resulted in a release of a compound with molecular mass about 30 kDa reacting with anti-MMP-14 and anti-MMP-15 antibody. Those compounds might be a soluble fragment of respective MT-MMP (data not published). 

Table 1 summarizes MMP content in control and pre-eclamptic umbilical cord tissues.

## 4. Conclusions

Human umbilical cord tissues contain matrix metalloproteinases. The MMPs and their tissue inhibitors (TIMPs) are engaged in metabolism of extracellular proteins resulting in the ECM remodelling of UC tissues. MMP-2 exerts the highest content in UC vessels. Its amount is several times higher than other MMPs. Gelatinase A is the main collagenolytic enzyme of both kind of UC vessel wall. 

Pre-eclampsia causes a decrease in MMP-2 content in UCAs and UCV. On the other hand, pre-eclamptic UC tissues exert changes in the content of other gelatinase, collagenase, matrilysins, and MT-MMPs, but their influence on protein metabolism in ECM seems to be minimal. We expected that reduced activity of collagen-degrading enzymes might be a factor, which enhances the accumulation of collagen and some other proteins in the UC tissues. 

Matrix metalloproteinases may be regulated by humoral factor(s) circulating in the umbilical cord plasma. It was described that these factors (especially IGF-1) are regulators of collagen and other proteins metabolism. IGF-1 has been found to stimulate collagen synthesis [64–67] and to inhibit the expression of MMP-1, MMP-3, MMP-8, and MMP-13 [64].

There are some difficulties and/or limitation in studying MMPs including separated determination of active and latent forms of those enzymes in umbilical cord and other tissues. Up to date, there are not commercially provided specific substrates for particular MMP activity measurement. Zymographic technique can be used only for those MMPs (like MMP-2 and MMP-9) that are present in high enough amounts. Low amounts of MMPs cannot be visualized by that method. Lack of commercial ELISA kits as well as lack of specific antibodies for detection of latent/active form by Western immunoblot technique or for self-made ELISA kits also limits the study. Because of those limitations, it is difficult to evaluate unequivocally their functional activities.

It seems to be possible that similar alterations occur in other fetal blood vessels. It may result in an increase in peripheral resistance as well as an increase in the blood pressure in the fetal vascular system. Some observations suggest that the raised pressure may persist after birth [68]. One may expect that the pre-eclampsia associated alterations of metalloproteinases activity in blood vessel walls may affect the vascular system in adulthood. Pre-eclampsia may be a factor that evokes an initiation of hypertension in utero and its amplification through childhood and adult life.

Future investigations can be focused on engagement of other enzymes important in remodeling of extracellular matrix of umbilical cord tissues, like cysteine proteases, serine proteases, and other metalloproteinases, for example, ADAMTS/ADAM metalloproteinases.

## Figures and Tables

**Table 1:** Matrix metalloproteinases of umbilical cord tissues in pre-eclampsia (*n* = 10, *P < .05, **P < .01, ***P < .001).

Matrix metalloproteinase	Umbilical cord artery		Umbilical cord vein		Wharton's jelly	
	control	Pre-eclamptic	control	pre-eclamptic	control	pre-eclamptic
collagenase						
MMP-1 [mg/kg protein]	5.76	3.19***	3.72	4.47*	—	—
stromelysin						
MMP-3 [mg/kg protein]	1.08	1.06	0.602	0.778**	—	—
gelatinase						
MMP-2 [mg/kg protein]	75.4	48.0***	54.8	37.6***	—	—
MMP-9 [mg/kg protein]	0.444	0.639***	0.419	0.708***	—	—
matrilysin						
MMP-7 [mg/kg protein]	0.258	0.365***	0.439	0.512*	1.663	1.682
MMP-26 [% of control]	100	92.4	100	72.7**	100	128.7**
Membrane-Type MMP						
MT-MMP-1 [% of control]	100	174***	100	157***	100	53***
MT-MMP-2 [% of control]	100	309***	100	217***	100	183***
MT-MMP-3 [% of control]	100	52***	100	60***	100	299***
MT-MMP-5 [% of control]	100	107	100	240***	100	94
